# Movement artefact removal from NIRS signal using multi-channel IMU data

**DOI:** 10.1186/s12938-018-0554-9

**Published:** 2018-09-10

**Authors:** Masudur R. Siddiquee, J. Sebastian Marquez, Roozbeh Atri, Rodrigo Ramon, Robin Perry Mayrand, Ou Bai

**Affiliations:** Florida International University, Miami, FL 33174 USA

**Keywords:** NIRS, Near infrared spectroscopy, Motion artefacts, Multi-channel IMU, Accelerometer, Gyroscope, Magnetometer, Artefacts removal, SNR improvement, De-noising

## Abstract

**Background:**

The non-invasive nature of near-infrared spectroscopy (NIRS) makes it a widely accepted method for blood oxygenation measurement in various parts of the human body. One of the main challenges in this method lies in the successful removal of movement artefacts in the detected signal. In this respect, multi-channel inertia measurement unit (IMU) containing accelerometer, gyroscope and magnetometer can be used for better modelling of movement artefact than using accelerometer only, which as a result, movement artefact can be more accurately removed.

**Methods:**

A wearable two-channel continuous wave NIRS system, incorporating an IMU sensor which contain accelerometer, gyroscope and magnetometer in it, was developed to record NIRS signal along with the simultaneous recording of movement artefacts related signal using the IMU. Four healthy subjects volunteered in the recording of the NIRS signals. During the recording from the first two subject, movement artefacts were simulated in one of the NIRS channels by tapping the photodiode sensor nearby. The corresponding IMU data for the simulated movement artefacts were used to estimate the artefacts in the corrupted signal by autoregressive with exogenous input method and subtracted from the corrupted signal to remove the artefacts in the NIRS signal. Signal-to-noise ratio (SNR) improvement was used to evaluate the performance of the movement artefacts removal process. The performance of the movement artefacts estimation and removal were compared using accelerometer only, accelerometer and gyroscope, and accelerometer, gyroscope and magnetometer data from IMU sensor to estimate the artefact in NIRS reading. For the remaining two subjects the NIRS signal was recorded by natural movement artefacts impact and the results of artefacts removal was compared using accelerometer only, accelerometer and gyroscope, and accelerometer, gyroscope and magnetometer data from IMU sensor to estimate the artefact in NIRS reading.

**Results:**

The quantitative and qualitative results revealed that the SNR improvement increases with the number of IMU channels used in the artefacts estimation, and there were approximately 5–11 dB increase in SNR when nine channel IMU data were used rather than using only three channel accelerometer data only. The artefact removal from natural movements also demonstrated that the combination of gyroscope and magnetometer sensors with accelerometer provided better estimation and removal of the movement artefacts, which was revealed by the minimal change of the HbO_2_ and Hb level before, during and after movement artefacts occurred in the NIRS signal.

**Conclusion:**

The movement artefacts in NIRS can be more accurately estimated and removed by using accelerometer, gyroscope and magnetograph signals from an integrated IMU sensor than using accelerometer signal only.

## Background

The absorption coefficient by human tissue in the near-infrared light region of 700–1000 nm [1] is much lower than other wavelength lights in the spectrum that are harmless to human body. This property leads to the development of near-infrared spectroscopy (NIRS) as a widely used method for detecting oxy- and deoxyhemoglobin level in blood. The NIRS signal can be detected by illuminating the human body with light from the near-infrared region [2, 3]. Moreover, the non-invasiveness, safety and cost-effectiveness [4], make NIRS even more popular than any other methods of detecting blood oxygenation level. The level of blood oxygenation in various parts of the human body convey a great deal of information about various physiological phenomena and processes [5], such as cardiovascular disease [6] and sepsis [7] from muscle oxygenation, cognitive involvement [8, 9] and activation of brain function [10, 11] from cerebral oxygenation.

Movement artefact removal is one of the most challenging parts of any type of bio-signal processing and the NIRS for blood oxygen level detection is no exception. It is not possible to fully restrict a subject from movement, voluntary or involuntary, and thus the acquired bio-signals are contaminated by the movement artefacts in different extents. Sometimes this contamination is so prominent that the subtle changes correspond to physiological changes subdued by the artefacts, thus the usability of the acquired signal mostly depends on the successful removal of the movement artefacts [12]. In the NIRS, the light source and the detectors are directly coupled to the human skin and this coupling is easily altered [13] by movement artefacts, which result in coupling error [14]. This coupling error imposes high uncertainty in the detection of the true changes in the NIRS signal which corresponds to the change in the physiological phenomena [15]. Moreover, from the perspective of the frequency domain, the NIRS signal variation due to the physiological change and the changes due to the movement artefacts are closely overlapped with each other, which makes it harder to separate the movement artefact content from the signal.

Numerous artefacts removal techniques for NIRS have been developed in the last few decades [16–22]. Most of them use the nature of the signal itself and the theoretical assumption of the influence of the movement artefact on the detected signal. A Majority of these methods development direction lie in the fact that detecting any other signal highly correlated to the movement artefacts was not readily available or difficult [23]. But the recent improvements in the Microelectromechanical systems (MEMS) chip components that is capable of registering motion information, i.e. acceleration, yaw, pitch, roll etc., makes it possible to observe motion-related signal concurrently with bio-signals of interest for various biomedical applications [24–29]. With respect to NIRS signal detection, this advancement in MEMS chip component makes it possible to record movement artefacts related motion data and the NIRS signal simultaneously [30–32]. This additional information related to movement artefact leads to the more effective use of the adaptive noise cancellation technique on NIRS signal. So far, to the best of our knowledge, all the research articles related to adaptive filtering to remove movement artefacts from NIRS signal uses only the three-channel accelerometer data to estimate the movement artefacts [25, 30, 31, 33]. In this study, nine-channel Inertia Measurement Unit (IMU) data, namely accelerometer, gyroscope and magnetometer, are used to estimate the movement artefacts in NIRS signal and subsequently to remove the motion-related movement artefact. The basis of the study is that, the more information correlated to movement artefacts available, the better the estimation of the interfering artefacts contribution on the detected signal, and greater SNR improvement can be achieved. Movement artefacts arise from diverse body movement, which might have very less acceleration but more rotational or directional change, would be better registered by gyroscope and magnetometer, and would result in better estimation of the movement artefacts.

Movement artefacts removal techniques commonly employ the autoregressive modelling (AR) to remove the artefacts from the NIRS signal [22, 34]. In this study, Autoregressive model with exogenous input (ARX) was used as the method to estimate the movement artefacts and the multi-channel IMU data served as the exogenous input to the system.

Adaptive noise cancellation (ANC) was widely used in the various field for noise cancellation [35]. In this approach, one or more additional channels of information that is highly correlated to the interfering noise component in the primary recorded signal is used to remove the noise. The ARX modelling can be assumed as an altered method of ANC, which applies the classical least-squares (LS) algorithm to estimate the noise using exogenous input as the reference source for indirect noise estimation in the observed signal. ARX modelling is extensively used in the problems related to system identifications. In the current study, IMU signals were used as the inputs to the ARX modelling to estimate the movement artefacts in the NIRS signal and then subtracted to estimate the true NIRS signal.

## Methods

### Data acquisition method

In this study, data acquisition with a single wavelength and a dual wavelength NIR light source were used during the recording from the subjects. The methodology presented in [16] was used to quantitatively assess improvement in the signal quality after the artefacts removal. The methodology requires that two version of the same NIRS channels are positioned as close as possible where one is impacted by movement artefacts and another remains unimpacted. In this respect, the unaffected signal is analogous to the “ground truth” signal presented in [16], which was denote here as “reference ground truth” as the actual “ground truth” cannot be acquired. For the first two subjects the single wavelength LED was used with simulated artefacts, and signal quality improvement in respect to SNR and correlation was used for quantitative assessment. For the remaining two other subjects, a dual wavelength NIR light source was used to record the data with actual movement of the subjects causing the movement artefacts. As the NIRS signal from the last two subjects were recorded using two wavelengths, the NIRS signals can be converted to blood oxygenation concentration changes using the modified Beer–Lambert law [36, 37] and the result of artefact removal can be compared to the expected hemodynamic changes. Considering that the hemodynamic change is minimal during a short duration when movement occurs, the levels of oxygenated (HbO_2_) and deoxygenated (Hb) hemoglobin concentration will be stable before and after movement occurs. Based on this, after the movement artefact removal from the contaminated portion of the NIRS signal, a minimal hemodynamic change with stable HbO_2_ and Hb levels is expected.

### Sensor system

To accomplish the above mentioned NIRS signal acquisition along with the simultaneous recording of multi-channel IMU data, a custom-made wearable NIRS system, based on Texas instrument (TI) CC3200 chip, was developed incorporating other peripheral chips and using the sensor node architecture developed in [38]. The IMU chip used in this architecture is MPU9250 which has 3-axis accelerometer, 3-axis gyroscope and 3-axis magnetometer data acquisition capability with a 16-bit resolution for each channel and the chip was attached to the NIR detector for better registration of the movement artefacts impact at the detector. In this study, each IMU channel was sampled at 62.5 Hz. An 850 nm wavelength LED and another 850–770 nm dual wavelength LED are used as the source of NIR light, and as the detector, a photodiode chip from TI modelled as OPT101 was used, which incorporate the required trans-impedance amplifier in the same chip. To digitize the analog signal from OPT101, a high precision ADC chip from TI, with the part no ADS1292, was used which has 24-bit resolution and high CMMR and support two-channel input. This ADC support multiple sampling rate ranging from 860 to 8000 Hz and we sampled the NIRS signal at 1000 Hz. To minimize the bus contention by the two vital peripheral devices in the system, IMU and ADC, these devices were connected to the main processor unit via the different buses, I2C and SPI respectively. The main processor unit used in this sensor architecture, CC3200, house two separate MCU in the same chip; one is featuring Wi-Fi Internet-On-a-Chip and another one as a typical microcontroller. Thus, the system can simultaneously collect the data from the peripheral devices and transfer those wirelessly through the internet to remote system or any local computer connected to the system using Wi-Fi.

### ARX modelling and artefacts removal

**Fig. 1:**
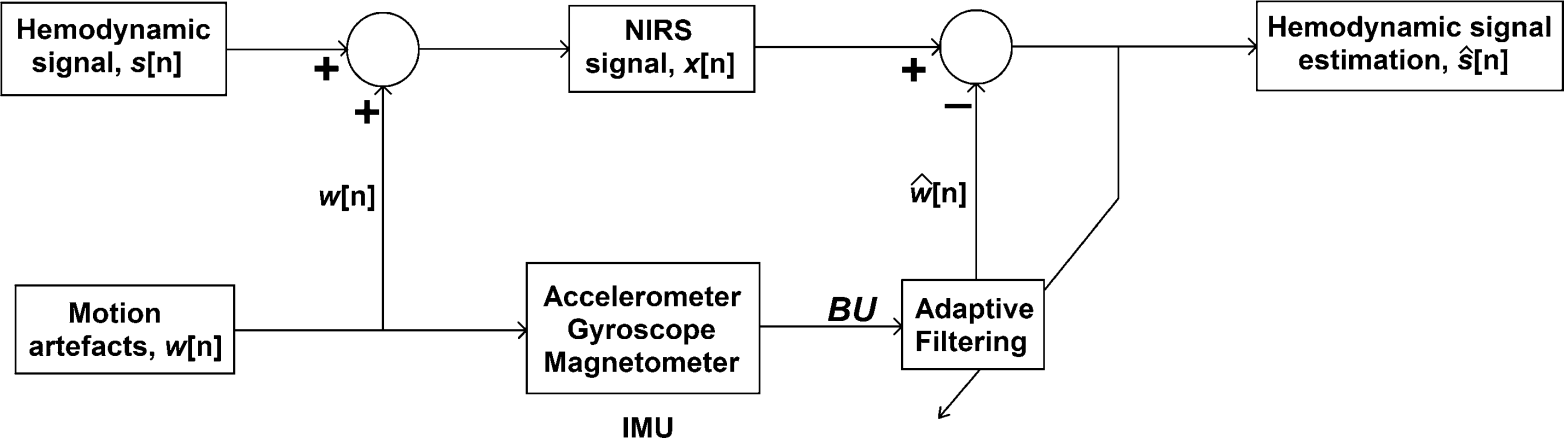
Block diagram of artefacts removal process

The artefacts estimation and removal process is outlined in Fig. 1. Let $s\left[ n \right]$ denote the true hemodynamic signal which was distorted by the motion artefacts signal $w\left[ n \right]$. This corrupted hemodynamic signal $x\left[ n \right]$ detected by the NIRS sensor can be expressed as,

1 $$ x\left[ n \right]\, = \,s\left[ n \right]\, + \,w\left[ n \right] $$

We used ARX modelling to estimate the motion artefacts in the detected signal. It is a widely used method in system identification task to determine the model structure using the input–output data. In this respect, it uses the least squares method to estimate the best set of the coefficient of the system model from the input–output data available. In our study, the system resembled a multiple input single output (MISO) model, because the IMU data inputted into the model consisted of multiple channel data. In this case the ARX modelling can be represented using the following equation,

2 $$ \hat{w}\left[ k \right]\, = \,a_{1} x\left[ {k - 1} \right]\, + \,a_{2} x\left[ {k - 2} \right]\, + \, \cdots \, + \,a_{NA} x\left[ {k\, - \,NA} \right]\, + \,\varvec{b}_{0}^{\varvec{T}} \varvec{u}\left[ k \right]\, + \,\varvec{b}_{1}^{\varvec{T}} \varvec{u}\left[ {k - 1} \right]\, + \, \cdots \, + \,\varvec{b}_{{\varvec{NB}}}^{\varvec{T}} \varvec{u}\left[ {k\, - \,NB} \right] $$

where $x\left[ n \right]$ is the detected NIRS signal and $\varvec{u}\left[ k \right]$ is the IMU data. Here $\hat{w}\left[ n \right]$ is the output from the system based on the model coefficients $\varvec{a} = \left[ {a_{1} \varvec{ }a_{2} \varvec{ } \cdots \varvec{ }a_{NA} } \right]$ and $\varvec{B} = \left[ {\varvec{b}_{0} \varvec{ b}_{1} \cdots \varvec{b}_{{\varvec{NB}}} } \right]$ when the input to the model is $\varvec{U} = \left[ {\varvec{u}\left[ k \right]\varvec{ u}\left[ {k - 1} \right] \cdots \varvec{u}\left[ {k - NB} \right]} \right]$. The model coefficients $\varvec{a}$ and $\varvec{B}$ selection in ARX modelling can be depicted by the following equation which is also known as least square method,

3 $$ J\left( {\varvec{a},\varvec{B}} \right)\, = \,\mathop \sum \limits_{k = 1}^{N} \left( {x\left[ k \right]\, - \,\hat{w}\left[ k \right]} \right)^{2} $$

where $\varvec{b}_{0}^{\varvec{T}} \varvec{ b}_{1}^{\varvec{T}} \cdots \varvec{b}_{{\varvec{NB}}}^{\varvec{T}}$ are $1\, \times \,L$ coefficient vectors and $\varvec{u}\left[ k \right]$ is a $L \times 1$ input vector, and the dimension $L$ is the number of IMU data channel used. There were three combination cases for $\varvec{U}$ used in this study, and they were,

Case 1: $$ \varvec{u}\left[ k \right]\, = \,\left[ {A_{x} \left[ k \right]\varvec{ }A_{y} \left[ k \right]\varvec{ }A_{z} \left[ k \right]} \right]^{T} ; \quad L\, = \,3 $$
Case 2: $$ \varvec{u}\left[ k \right]\, = \,\left[ {A_{x} \left[ k \right]\varvec{ }A_{y} \left[ k \right]\varvec{ }A_{z} \left[ k \right]\varvec{ }G_{x} \left[ k \right]\varvec{ }G_{y} \left[ k \right]\varvec{ }G_{z} \left[ k \right]} \right]^{T} ; \quad L = 6 $$
Case 3: $$ \varvec{u}\left[ k \right]\, = \,\left[ {A_{x} \left[ k \right]\varvec{ }A_{y} \left[ k \right]\varvec{ }A_{z} \left[ k \right]\varvec{ }G_{x} \left[ k \right]\varvec{ }G_{y} \left[ k \right]\varvec{ }G_{z} \left[ k \right]\varvec{ }M_{x} \left[ k \right]\varvec{ }M_{y} \left[ k \right]\varvec{ }M_{z} \left[ k \right]} \right]^{T} ;\quad L = 9 $$

For the conventional studies [22, 30, 32, 33] where only three channels Accelerometer data are used for autoregressive modelling, $\varvec{U}$ can be expressed as in case 1 where *A*_*x*_*, A*_*y*_ and *A*_*z*_ represent the three channels of Accelerometer data. Additional to the conventional study using only three channels Accelerometer data, we extended the study using six channels and nine channels IMU data. In case 2 and 3, *G*_*x*_*, G*_*y*_ and *G*_*z*_ represent the three channels of Gyroscope data and *M*_*x*_*, M*_*y*_ and *M*_*z*_ represent the three channels of magnetograph data.

In the artefact estimation process, the portion of the NIRS signal containing movement artefacts was fed to the ARX modelling algorithm as the output and the corresponding multichannel IMU data as the input to determine the model coefficients. For each artefact segment, this operation was iterated using several combinations of model orders from 1 to 10 and the order selected for who’s the sum of squared error was the minimum between the estimation and the detected signal. Using the returned coefficient, a simulation model was defined for the current noisy portion of the NIRS signal. In the newly defined simulation model, the IMU signals were used as the input to the model to get the estimation $\hat{w}\left[ n \right]$. Here, the estimation closely resembled to artefact $w\left[ n \right]$ as the signal contribution by the true hemodynamic signal $s\left[ n \right]$ to the detected signal $x\left[ n \right]$ was very small compared to the artefact’s contribution. Thus this estimated signal $\hat{w}\left[ n \right]$ was then subtracted from the observed signal $x\left[ n \right]$ to get the estimation of the movement artefacts free hemodynamic signal $\hat{s}\left[ n \right]$ as per the equation,

4 $$ \hat{s}\left[ n \right] = x\left[ n \right] - \hat{w}\left[ n \right] $$

### SNR improvement

As the data acquisition methodology used in this study was very much similar to [16], the SNR calculation was also done using similar formula applied in that study. The difference in the SNR, before and after the artefacts removal, is calculated using the following equation which is described in [39],

5 $$ \Delta SNR\, = \,10 log_{10}^{{\left( {\frac{{\sigma_{x}^{2} }}{{\sigma_{{e_{after} }}^{2} }}} \right)}} \, - \,10 log_{10}^{{\left( {\frac{{\sigma_{x}^{2} }}{{\sigma_{{e_{before} }}^{2} }}} \right)}} $$

where, $\sigma_{x}^{2}$ is the variance of the movement artefacts free signal which is the referenced ground truth and $\sigma_{{e_{before} }}^{2}$ and $\sigma_{{e_{after} }}^{2}$ are the variance of the signal with the movement artefacts before and after the artefacts are removed, respectively.

### Subjects and experimental design

Four healthy subjects, ages 22, 25, 27 and 28, with no history of asphyxia or brain disorder, volunteered for this study and a total of twelve sessions of data were collected. The NIRS were recorded from the forehead for simplicity. All the subjects were instructed to sit comfortably during the NIRS recording. As mentioned before, the NIRS signals from the first two subjects were contaminated by simulated movement artefacts. These simulations were done by external tapping on one of the two optical sensors, while the other remained unaffected. The NIRS signal from the other two subjects were collected by dual wavelength NIR light source, the subjects were instructed to move their head to induce natural movement artefact in the NIRS signals.

## Results

**Fig. 2:**
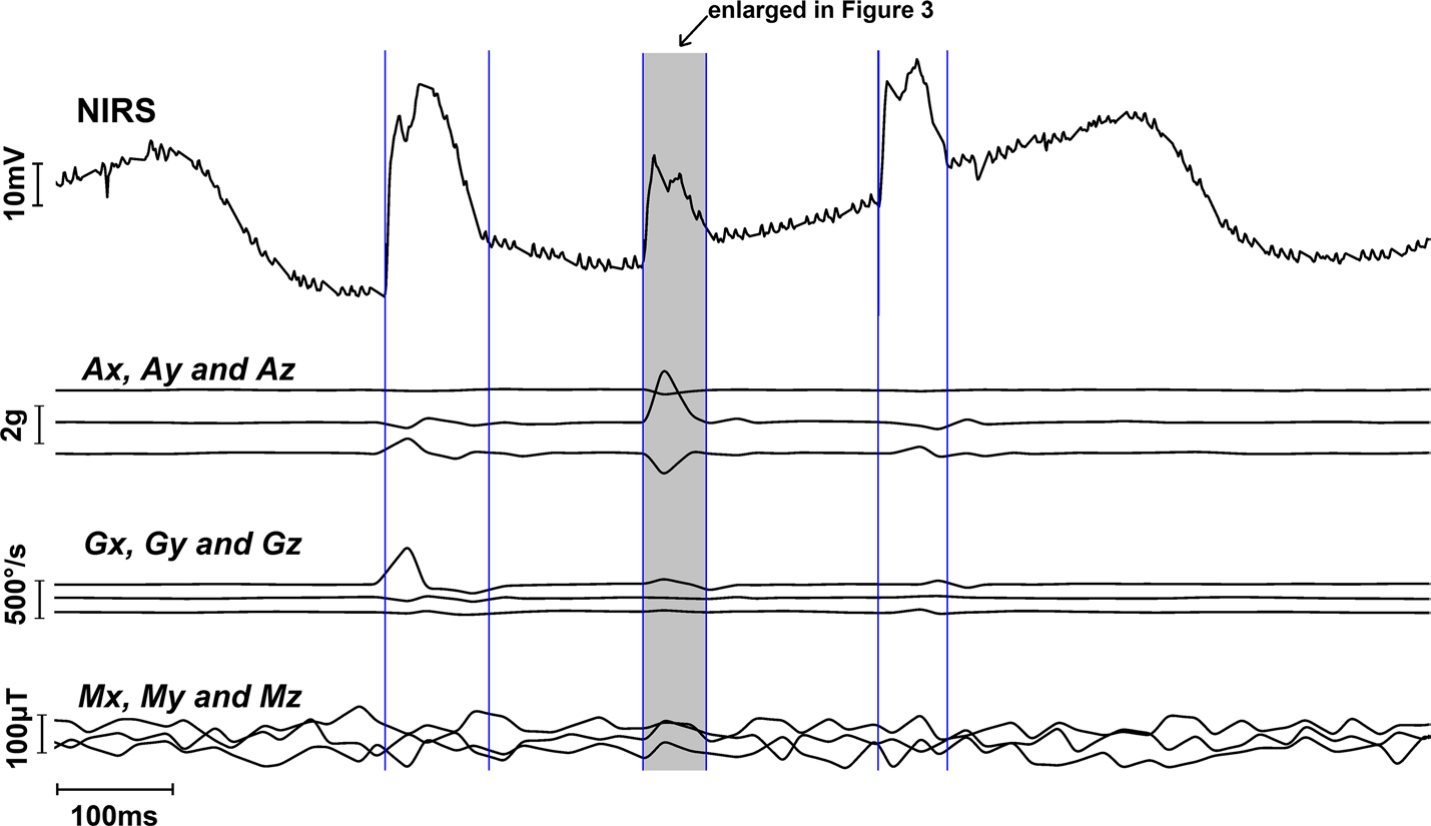
Raw data of one channel NIRS signal containing 3 noise segments indicated by vertical blue lines and corresponding 9 channel IMU signals

In this study, the artefacts removal from the NIRS signal was implemented on the raw signal from the optical sensor. Thus, the clean signal (after the movement artefact removal) can be used in any other processing for further study. Figure 2 depicts a representative portion of the raw NIRS and the corresponding IMU data from subject 1. This NIRS signal contains three movement artefact segments which are indicated by vertical lines in the topmost plot in the figure. The simultaneous IMU data of this portion, which are three channels accelerometer, three channels gyroscope and three channels magnetometer data, are plotted in the same figure. It is already mentioned earlier that most of the IMU-based movement artefact removal studies used three channels accelerometer data [30, 31, 33], whereas in this study, it was observed from the raw signal that without any processing, the Gyroscope data have the most impact from the motion artefacts and thus highly effective in estimating the artefacts. In the signal portion presented in Fig. 2, the gyroscope data have a more prominent impact on all three movement artefact segments in comparison to the accelerometer and the magnetometer data, which is apparent in Fig. 2.

**Fig. 3:**
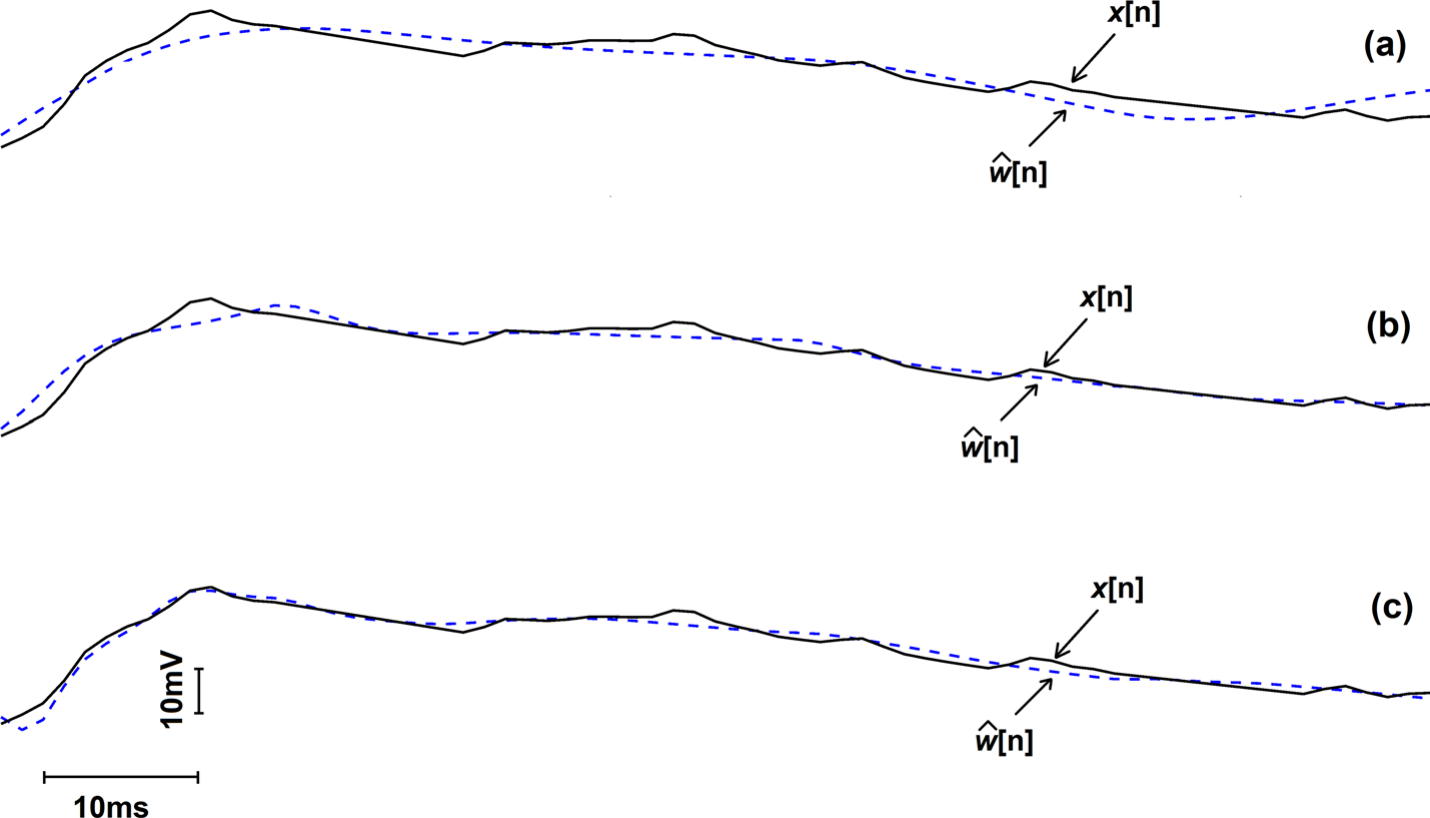
The estimated noise signal (blue dotted line) and the original signal (black solid line) for the second noise segment depicted in Fig. 2. Plot (a) shows the result when 3 channels were used, plot (b) when 6 channels were used and plot (c) when 9 channels were used

The movement artefact estimation for the second segment in Fig. 2 is presented in Fig. 3. In Fig. 3, movement artefact containing signal is depicted by the solid black line, and the estimation of the movement artefact is indicated by the dashed blue line. Three estimation results are presented qualitatively in Fig. 3; the plot (*a*) for the case when only accelerometer data is used to estimate, the plot (*b*) presents the estimation result when accelerometer and gyroscope data are used in the modelling and lastly bottom-most plot (*c*) shows the estimation result when all the nine-channel IMU data, namely accelerometer, gyroscope and magnetometer, were used. This qualitative representation indicates that the more IMU channels that were used to model, the better the estimation was, and the best estimation was achieved when all the nine channels of IMU data available were used.

The quantitative metric, to assess the performance of the artefacts removal technique used in this study was the improvement in SNR which was calculated according to the Eq. (5) described in the previous section. This same metric and calculation was also used in several other research related to artefacts removal techniques presented in [16, 22, 32, 39]. In this respect, Table 1 represents the data of SNR improvement’s quantitative data for the three movement artefact segments indicated in Fig. 2, which belongs to the data recorded from the subject 1 and for the data from the subject 2. For all the six segments, SNR improvements have been calculated in the cases of using three channels (only accelerometer data), six channels (accelerometer and gyroscope data) and nine channels (accelerometer, gyroscope and magnetometer). The data presented in Table 1 indicates that for all the movement artefact segments, SNR improvements are higher when gyroscope and magnetometer data are used along with the accelerometer data. Specifically, for the first movement artefact segments from subject 1, we had 11.33 dB SNR when six channels were used, which was 3.41 dB higher than the SNR when only three channels were used, and the SNR was 14.77 dB when nine channels were used which was 6.85 dB higher than the SNR we got for three channels only. Similarly, for the second movement artefact segment, we had 15.11 dB SNR which was 6.18 dB higher than the SNR when we only used three channels IMU data and for the third movement artefact segment, we had 13.92 dB SNR which was 8.61 dB higher than the result for the three channels only case. Additionally, we computed the correlation between the artefacts removed signal and reference ground truth signal for each movement artefact segments which are also presented in Table 1. For the first movement artefact segment, the correlation coefficient was increased from 0.79 to 0.82 when six channels IMU data were used to remove the movement artefacts than when only three channels data were used, and this value increased to 0.83 when nine channels IMU data were used. In case of the second movement artefact segment, the correlation coefficient was increased from 0.89 to 0.92 and 0.93 when six channels and nine channels IMU data were used respectively to remove the movement artefacts contribution in the signals. For the third movement artefact segment the correlation between the movement artefact removed signal and the reference ground truth signal was 0.94 when only three channel IMU data were used to remove the artefacts and it was 0.96 when six or nine channel IMU data were used for artefacts removal.

**Table 1:** SNR improvement of 6 representative segments of noise in the recording from the subject 1 and 2, when various number of IMU channel data were used to remove the artefacts

Subjects	Accel.		Accel. + Gyro.		Accel. + Gyro. + Magn.	
	SNR (dB)	Correlation	SNR (dB)	Correlation	SNR (dB)	Correlation
Sub 1 Seg 1	7.92	0.79	11.33	0.82	14.77	0.83
Sub 1 Seg 2	8.93	0.89	12.66	0.92	15.11	0.93
Sub 1 Seg 3	5.31	0.94	13.92	0.96	13.92	0.96
Sub 2 Seg 1	7.35	0.96	13.44	0.97	19.08	0.97
Sub 2 Seg 2	3.04	0.77	5.29	0.78	11.95	0.98
Sub 2 Seg 3	11.56	0.94	14.31	0.95	17.46	0.95

The correlation coefficient presented in the table is between artefacts removed signal and the ground truth signal

In the data recorded from the second subject, another three movement artefact segments were selected and all the similar processing described above were applied on those movement artefact segments. The quantitative SNR improvements for those segments are also presented in the data Table 1 which shows the similar trend in the SNR improvements for subject 1. In case of movement artefact segments from the subject 2, we had 19.08 dB SNR when we used nine channels IMU data which was 11.73 dB higher than the result for three channels usage case for the first movement artefact segment. Likewise, for the second movement artefact segment from subject 2, we had 11.95 dB SNR and for third segment 17.46 dB when nine channels IMU data were used which were 8.91 and 5.90 dB higher than when six and nine channels IMU data were used, respectively. In respect of correction coefficient, the first movement artefact segment for this subject had a coefficient of 0.96 between movement artefact removed signal and referenced ground truth signal when three channels IMU data were used and it 0.97 when six or nine channels IMU data were used. For the second movement artefact segment, the correlation coefficient was increased from 0.77 to 0.78 and 0.99 when we increased the number of IMU channels used in artefact removal to six and nine channels respectively from only three channels data. The last movement artefact segment from this subject had a correlation coefficient of 0.94 between the movement artefact removed signal and the reference ground truth when three channels IMU data were used to remove movement artefacts and 0.95 when six or nine channel IMU data were used.

**Fig. 4:**
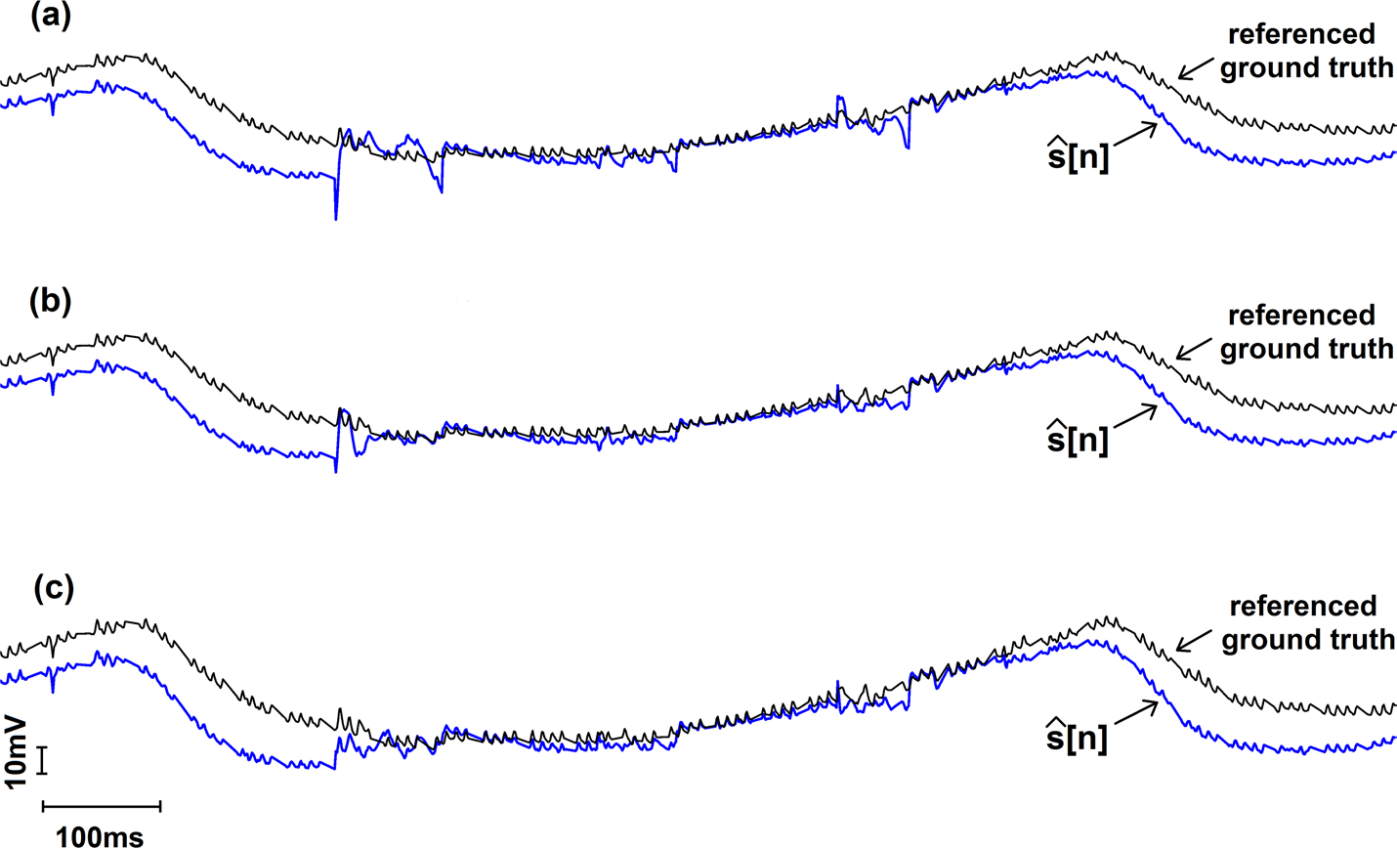
Qualitative representation of the NIRS signal after replacing the 3 artefacts containing segments by de-noised signal along with the referenced ground truth signal from the other NIRS channel which is analogous to “ground truth” signal. Plot (a) shows the result when three channels were used, plot (b) when six channels were used and plot (c) when nine channels were used

The qualitative result of removing the artefacts that contaminated the abovementioned three segments present in the recording from subject 1 is presented in Fig. 4 which are plotted with the solid blue lines, whereas the artefacts-free NIRS signals from the other channel are also concurrently plotted in the same figure using black solid lines to indicate the empirical comparisons. Similar to the presentation used in Fig. 3, the result of a various number of IMU channel data usage is presented in Fig. 4, plot (*a*) represent the result when only accelerometer data were used, plot (*b*) when accelerometer along with the gyroscope data were used and finally plot (*c*) depicts the result when accelerometer, gyroscope and magnetometer data were used altogether.

In case of quantitative results, it was easy to determine the best outcome of the artefacts removal as it was obvious from exact values of SNR presented in the data table that the higher the SNR, the better the result is. But in case of empirical result, there is no direct way to determine the best. In this respect, in the current study, we assumed the artefact-free NIRS channel to be the reference ground truth signal, to compare empirically. Empirically, we can say that the closer the variance of NIRS signal after the artefacts removal to the variance of the referenced ground truth signal, the better the result is. In that sense, we can say the best result was achieved when nine channels of IMU data were used, which is also consistent with SNR improvement data presented in Table 1 and apparent from the plot (*c*) in Fig. 4.

**Fig. 5:**
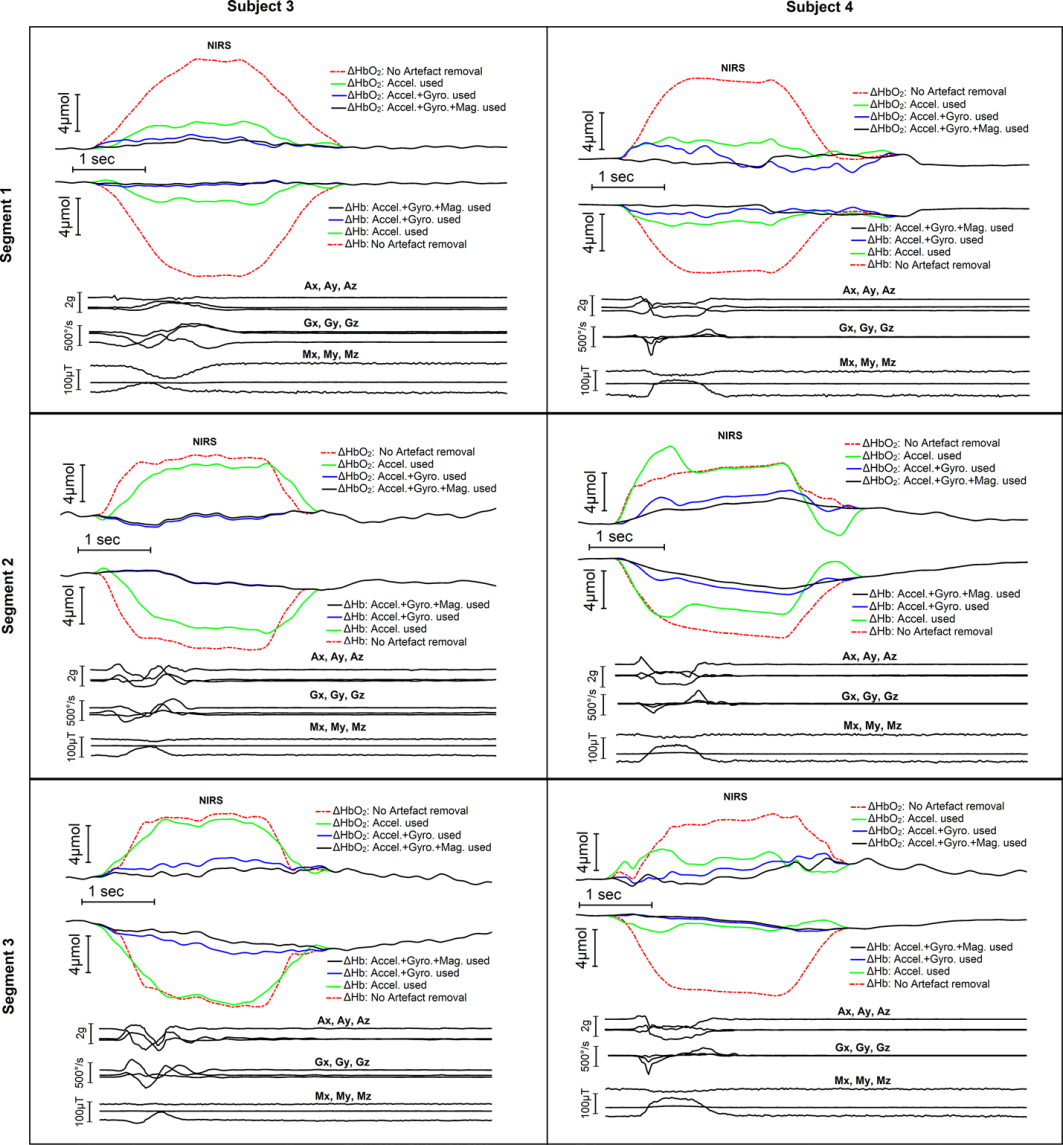
Effect of estimating and removing movement artefacts using multi-channel IMU signals on HbO_2_ and Hb change detection

The NIRS signal from the subject 3 and 4 were recorded using dual wavelength NIR light source, so that it can be converted to oxygenated (HbO_2_) and deoxygenated (Hb) hemoglobin concentration changes with typical processing. The movement artefacts were removed from the raw NIRS signal using IMU signals as described in the previous section and then converted the denoised signals to HbO_2_ and Hb changes using typical Beer–Lambert law [36]. Three noise segments data from each of the two subjects are presented in Fig. 5. Each of the six plots in Fig. 5 present the HbO_2_ and Hb change when no denoising was used, when only accelerometer signals were used, when accelerometer and gyroscope signal were used and when accelerometer, gyroscope and magnetometer signal were used for artefacts estimation and removal. In all the plots there were substantial changes in HbO_2_ and Hb concentration around the movement artefacts containing parts of the signals when no denoising were used. For all the six artefact segments, HbO_2_ and Hb changes curve get closer to the minimal change when artefacts estimation and removal was done for the artefacts containing part of the NIRS signal, and the case of estimating the artefacts by accelerometer, gyroscope and magnetometer result better than the case of using only accelerometer for the estimation and removal of the movement artefacts.

## Discussion

In this paper, a method of using multi-channel IMU data to successfully remove movement artefacts from NIRS signal has been presented. The qualitative and quantitative results show that implementation of multi-channel IMU data resulted in more accurate modelling of motion artefacts in NIRS, and thus we obtained more accurate motion artefacts free NIRS signal which can be used to detect physiological changes accurately.

After the first massive production of micro-electromechanical-system (MEMS) chip based accelerometer in about 1993 [40] it was extensively used in other fields such as automobile, aerodynamics and so on; but there was a sloth progress in effectively using such devices in the field of bio-signal acquisition due to the reduction of comfort of the subject [32] which results from the extra wiring and placement of this additional sensors. Moreover, placing the accelerometer sensor close to the NIR sensor was also another challenge as the NIRS system used in most of the research, utilized the optical fiber based light transport system [41] to and from the subject body. This close placement of the accelerometer to the light coupling to the subject body is necessary to record motion artefacts caused by the subject movement as well as due to the NIR sensor shifting [32]. These challenges were mitigated in this study by careful selection of the components used in the design of the system and making the system as wearable as possible, for instance, the IMU chip, MPU9250, used in the system is only 3 mm by 3 mm in dimension and this IMU was attached to the body of the NIR detector sensor OPT101 chip to record the true motion of this detector as well as the movement of the subject body. The entire wireless NIRS system used in this study has a dimension of only 30 mm by 48 mm and power by a small lithium-ion battery, which makes it a true wearable NIRS data acquisition system. Considering the subtle details like those presented above could be helpful in designing wearable NIRS system incorporating sophisticated chip like IMU which will improve the overall system performance.

The main challenge related to the hardware of the system in this study came from the strategy used in the acquisition of the multi-channel NIRS signals. As already mentioned earlier, to use the effective method of quantitative evaluation of the result of movement artefacts removal success, the two NIRS detectors were placed as close as possible with the target of recording a very similar version of NIRS signal where one of them are intentional movements’ artefacts induced. Due to this closeness, inducing artefacts in one NIRS channel while leaving another channel undisturbed was tough and a lot of attempts took place to achieve this data acquisition strategic goal.

The effectiveness of the regressive modelling, like the ARX used in this study to estimate motion artefacts, highly depends on how much motion related information present in the exogenous input. The raw data presented in Fig. 2 has an important finding in this context, the first and the last artefact segments have a high impact on the gyroscope data whereas, for the middle artefact segment, the accelerometer data have a prominent impact. This finding implies that, using more channels of IMU data increases the probability of capturing the motion-related data in at least some of the channels which in turn increase successful estimation of the movement artefacts. In this respect, the SNR improvement results presented in Table 1 depicts that SNR improvement increased if the number of IMU data channel used in the modelling were increased except for the movement artefact segment 3 from the subject 1. For the movement artefact segment 3 from subject 1, the SNR improvement value for the six channel and nine channel IMU data, are same, which is due to the fact that the last three channel of the IMU data (the magnetometer data) had insignificant contribution on the artefact estimation. This insignificant contribution might have two reasons, either the artefact was indifferent to the variable the sensor was sensing or the sensor itself was not sensitive enough to detect the subtle change in that variable. On the other hand, for the movement artefact segment 2 from subject 2 in Table 1, the magnetometer data has a high contribution to the SNR improvement result. Besides the SNR improvement results, correlation coefficients between the movement artefact removed signal and the referenced ground truth signal are presented in Table 1 for both subjects 1 and 2 for each of the movement artefact segments as another quantitative improvement indicators. This indicator signifies how much alike the signals are in respect of covariance by a single unitless quantity ranging from − 1 to + 1 where values closer to + 1 indicates stronger correlation between the signals [42]. For all the movement artefact segments the correlation coefficients increase towards + 1 when six channels of IMU data were used than only when three channels IMU data were used to estimate and remove the movement artefacts. When nine channel IMU data were used to estimate and remove the movement artefacts, the correlation coefficients remained same for some of the segments which is analogues to the case of SNR improvements after artefact removal from the third movement artefact segment from the first subject.

The data from the subject three and four are presented in Fig. 5. In the experiments with these two subjects, the movement artefacts in the NIRS were induced by natural head movements rather than the simulated movement artefacts as presented for the first two subjects. Furthermore, the NIRS signals were converted into oxygenated (HbO_2_) and deoxygenated (Hb) hemoglobin change using Beer-Lambert law [36]. In contrast to the experiments with simulated artefacts where validation of the artefacts removal was assessed by the SNR and correlation improvement, the same validation method cannot be used in case of natural movement artefacts removal due to the lack of any reference ground truth signal. Considering that there was a minimal hemodynamic change during a short duration of the natural movement occurrence, a minimal change in concentration of the HbO_2_ and Hb was used to determine the performance of the artefacts removal associated with natural movements. The result presented in Fig. 5 demonstrates that with additional gyroscope sensors and magnetometer, the artefact in NIRS signals can be better removed as revealed by the minimal change of HbO_2_ and Hb signals before, during, and after the movement artefacts occurred. This suggests that, in addition to the accelerometer in the IMU sensor, gyroscopes and magnetometer in the IMU are complementary to the accelerometer for a better modelling of the movement artefacts, which as a result, leads to better removal of the movement artefact in NIRS signals. A previous study [21] showed that the level of negative correlation between Hb and HbO_2_ get reduced when movement occur. In case of this study, the duration of the natural head movement resulting artefact in NIRS signals was relatively short and hypothetically, there would not be any significant hemodynamic change during this short period. Based on this assumption, the distorted waveform of Hb and HbO_2_ signals as depicted in Fig. 5, was due to the artefacts contaminated in the NIRS signal. As the distorted Hb and HbO_2_ waveform was due to the artefacts, the distortion should be independent to the real hemodynamic changes of Hb and HbO_2_, which might be positively or negatively correlated. In the experiments performed in this study, the data showed a major negative correlation between the oxyhaemoglobin and deoxyhemoglobin signal; however, after removing the distortion induced by the artefact, the level of Hb and HbO_2_ kept the same as the baseline, which was in agreement with the hypothesis.

It is to be noted here, the selection of the movement artefact segments were done manually by keeping track of the time of movement artefact occurrence and later visual inspections on the raw IMU data and the raw NIRS signal. This manual detection of the segment will be automated in future based on IMU and NIRS signal feature changes. Though this study was not purposed to evaluate the quality of NIR signal from the custom-made system, we did perform preliminary evaluation—the raw NIRS signal was visually inspected for the presence of heart beating signal, showing whether the signal was correctly acquired. However, the accuracy and quality of the NIR signals using the custom-made system needs further well-controlled test, particularly, the correlation with brain functions. This will be the goal for the future study.

Although the current results presented in this paper showed a significant improvement in artefacts removal, there are still a lot of scopes to improve the developed technique. From the system identification point of view, any movement artefact impact on the NIRS signal is unpredictable in nature as they might differ in amplitudes, directivities, latencies, frequency contents and so on [41]; moreover, it has been observed in this research that various IMU channel might have different level of artefacts impact in different cases, which is another variability probably arise from sensor or from the nature of the artefact itself.

## Conclusion

In the previous studies, accelerometer was used in adaptive filtering for movement artefacts registering and estimating its impact in NIRS signal. The theoretical application to accelerometer-based motion artefact removal is effective in mechanical systems, but the organic movements of a human subject are not only subjected to linear movements, but simultaneous rotation and multi-directional displacements. These motions are roughly captured by the accelerometer but are effortlessly quantified with the use of the additional magnetometer and gyroscope. Thus, movement artefacts related signals detected by other sensors from IMU, along with the accelerometer signal, result in better estimation of the movement artefacts in the detected NIRS signal. In this study, the result showed that using the accelerometer, gyroscope and magnetometer signals from IMU sensor provide more accurate modelling of motion artefacts and thus improves the SNR improvement yields.
